# Development of an HPLC-PDA Method for the Determination of Capsanthin, Zeaxanthin, Lutein, β-Cryptoxanthin and β-Carotene Simultaneously in Chili Peppers and Products

**DOI:** 10.3390/molecules28052362

**Published:** 2023-03-03

**Authors:** Jiayue Xu, Jialu Lin, Sijia Peng, Haoda Zhao, Yongtao Wang, Lei Rao, Xiaojun Liao, Liang Zhao

**Affiliations:** 1 College of Food Science & Nutritional Engineering, National Engineering Research Center for Fruit and Vegetable Processing, Key Laboratory of Fruit and Vegetable Processing of Ministry of Agriculture and Rural Affairs, Engineering Research Center for Fruits and Vegetables Processing of Ministry of Education, Beijing Key Laboratory for Food Non-Thermal Processing, China Agricultural University, Beijing 100083, China; 2 Farm, Land and Agri Business Management Department, Harper Adams University, Newport TF10 8NB, UK

**Keywords:** chili pepper and products, carotenoids, HPLC-PDA, simultaneous determination, extraction

## Abstract

For the better standardization and widespread application of the determination method of carotenoids in both chili peppers and their products, this work reports for the first time the simultaneous determination of five main carotenoids, including capsanthin, zeaxanthin, lutein, β-cryptoxanthin and β-carotene in chili peppers and their products, with optimized extraction and the high-performance liquid chromatography (HPLC) method. All parameters in the methodological evaluation were found to be in good stability, recovery and accuracy compliance with the reference values; the *R* coefficients for the calibration curves were more than 0.998; and the LODs and LOQs varied from 0.020 to 0.063 and from 0.067 to 0.209 mg/L, respectively. The characterization of five carotenoids in chili peppers and their products passed all the required validation criteria. The method was applied in the determination of carotenoids in nine fresh chili peppers and seven chili pepper products.

## 1. Introduction

The production of chili peppers (*Capsicum annuum* L.), which are commonly grown as spice vegetables, reached 38.027 million tons in 2019 and climbed by 28.12% from 2010 [1]. A fresh chili pepper’s high concentration of vitamin C and E, calcium, phosphorus, iron, carotenoids and capsaicin made it the “greatest nutritional vegetable” [2], and chili products are widely available in the market and in commerce. Additionally, due to high added value, deep-processed chili products (such as carotenoids, capsaicin, etc.) have received a lot of attention with the rapid development of active ingredient extraction technology [2,3]. For instance, the carotenoids found in chili peppers, primarily capsanthin, capsorubin, β-carotene, zeaxanthin, β-cryptoxanthin, violaxanthin, lutein, and antheraxanthin [4] all have high biological activity, which has been thoroughly investigated. The primary carotenoid in chili peppers, capsanthin, is the most widely used pigment in nature. It has numerous health benefits for humans, including antioxidant properties, cancer- and radiation-fighting properties, immune modulation, regulation of body lipid metabolism, and the molecular prevention and treatment of chronic cardiovascular diseases [5,6]. Finding the added-value components, such as the primary carotenoids in chili peppers and their products, has become more important recently. However, the majority of investigations concentrated on the determination of carotenoids in either fresh chili peppers or chili pepper products [7,8,9]. There was no study that could be used to influence the trade and industry of chili peppers and their products by simultaneously determining the carotenoids in them. Additionally, the existing techniques for extracting and determining the carotenoids in chili peppers or chili pepper products were more difficult and inconvenient [10]. Therefore, it is required to develop a single practical extraction measurement technique for the carotenoids in chili pepper and their products.

Recent studies have focused on the five primary processes that go into determining carotenoids: sample pretreatment, extraction, saponification, separation and identification. To increase the effectiveness of extraction, sample pretreatment included sample drying (sun drying, air drying, freeze drying, osmotic dehydration and chemical dehydration) [11,12,13]; there are two types of extraction: traditional solvent extraction and new solvent extraction. The latter is more eco-friendly and effective and uses techniques such as microwave-assisted extraction [14], ultrasound-assisted extraction [15], super-critical fluid extraction [16] and enzyme-assisted extraction [17]; saponification is utilized in an alkaline solution to increase the quantification of carotenoids after eliminating chlorophyll and esterified lipids [18]; and high-performance liquid chromatography (HPLC) with a photo-diode array (PDA) detector is a popular and effective separation technique [19]. Mass spectrometry is employed as a detector to provide more precise information about the extract’s structure while correctly identifying each carotenoid [20]. 

This study focused on optimizing and establishing a practical method for the simultaneous determination of five main carotenoids, including capsanthin, zeaxanthin, lutein, β-cryptoxanthin and β-carotene, in industrialized and natural chili pepper and its products using HPLC. The method was validated by linear range, limit of detection and limit of quantification, stability, recovery and accuracy. This study also described the five carotenoids in nine fresh chili peppers and seven commercially available chili pepper products.

## 2. Results and Discussion

### 2.1. HPLC Condition Optimization

#### 2.1.1. Wavelength

The wavelengths of capsanthin, zeaxanthin, lutein, β-cryptoxanthin, and β-carotene in the 400–500 nm range were scanned, as shown in Figure S1. In 400–500 nm, the absorbance of capsanthin, β-carotene, zeaxanthin and lutein had a large absorption at around 450 nm, and β-cryptoxanthin had a large absorption at 470 nm, which was different from the maximum absorption wavelengths of the other four carotenoids and the theoretical maximum absorption wavelength, which might be related to the purity of the standards. In summary, combining the results of the current studies, the optimal wavelength was determined to be 450 nm [21,22].

#### 2.1.2. Mobile Phase Gradient

In Figure S2, the chromatograms of capsanthin, zeaxanthin, lutein, β-cryptoxanthin and β-carotene using six mobile phase gradient methods are shown. Resolution (R) was used to describe the degree of separation of different carotenoids: R = 1.0 instructed that the degree of separation could reach 98%; usually R ≥ 1.5 instructed that components could be completely separated [23,24]. Comparing several methods utilizing a five mobile phase gradient, method 6 was able to effectively separate the five carotenoids, with the R-value of β-cryptoxanthin being 1.49 and the R-values of the other four carotenoids being >3.5. The target peaks were all sharp and symmetrical, and there were no consecutive peaks, trailing peaks or forward peaks. They were evenly distributed in the middle part of the chromatogram, with high response values. The analysis time was appropriate and there were no solvent peaks and spurious peaks in the chromatograms. The results demonstrated that gradient 6 was ideal in terms of compatibility and peak generation, which were significant characteristics previously highlighted in other studies [7]. Additionally, a comparable gradient was examined in another study with apparent separation, but the targeted peak response was lower than our results and the running time was approximately twice as long as that in our study, further demonstrating the superior efficiency and advanced nature of our method [25].

#### 2.1.3. Mobile Phase

As shown in Figure S3, when the mobile phase was acetone–water, five carotenoids were well separated, with sharp and symmetrical target peaks and high response values. The analysis time was less than 18 min when the targeted peaks were positioned in the middle with improved separation. When the mobile phase was MeOH–water or acetonitrile–water, only β-cryptoxanthin and β-carotene were separated and the chromatogram had spurious peaks, and the peaks of capsanthin, zeaxanthin and lutein had incredibly low response values. Zeaxanthin, lutein and capsanthin could not be separated from each other when the mobile phase was tetrahydrofuran–water. As a result, acetone–water was the optimal mobile phase, which was also used in other studies to determine carotenoids in vegetables [26], but the running time for a comparable study to determine the content of carotenoids in chili pepper products was about 35 min, which was twice as long as our time [25].

#### 2.1.4. Flow Rate of Mobile Phase

According to Figure S4, all peaks were sharp and symmetrical with high response values under the three flow rates of the mobile phase. At the flow rate of 0.5 mL/min, the R-value of zeaxanthin and luteolin was 1.34, with a minor internal peak overlap and late peak appearance. At a flow rate of 1.0 mL/min, compared to a flow rate of 1.5 mL/min, the peak areas of the five carotenoids were significantly higher. In summary, 1.0 mL/min was selected as the mobile phase flow rate, which was also set as the typical fluid rate in earlier studies [9].

#### 2.1.5. Column Temperature

Carotenoids are extremely sensitive to temperature due to their linear and inflexible structure [27]. As shown in Figure S5, the shapes of the target peaks were sharp, symmetrical, and had high response values at each of the three column temperatures. Zeaxanthin and lutein had a slightly worse R-value of 1.47 at the 25 °C column. In addition, the analysis time was somewhat shortened with the rise in column temperature. The best column temperature was determined to be 30 °C in order to maintain the stability of carotenoids. In the study of Wissanee Pola [28], the greater carotenoid contents accumulated at 30 °C combined with considerably evaluated genes related to carotenoid biosynthesis, indicating that 30 °C would be the proper temperature for more carotenoid detection in HPLC.

**Figure 1:**
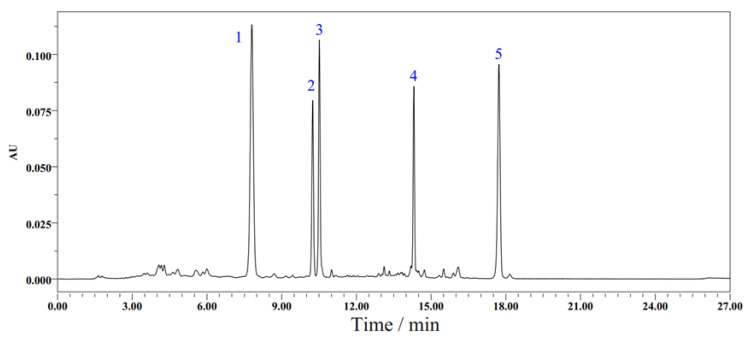
Chromatogram of capsanthin, zeaxanthin, lutein, β-cryptoxanthin and β-carotene under optimized HPLC method (1: capsanthin, 2: zeaxanthin, 3: lutein, 4: β-cryptoxanthin, 5: β-carotene).

In conclusion, the optimal HPLC conditions for determining five carotenoids in chili pepper (spectrum results as shown in Figure 1) were a column temperature of 30 °C, a detection wavelength of 450 nm, a sampling volume of 10 μL, a flow rate of 1 mL/min and mobile phases of acetone (solvent A) and water (solvent B), with an elution gradient of 0–5 min, 75% A; 5–10 min, 75–95% A; 10–17 min, 95% A; 17–22 min, 95–100% A, 22–27 min, 100–75% A.

### 2.2. Extraction Condition Optimization

**Figure 2:**
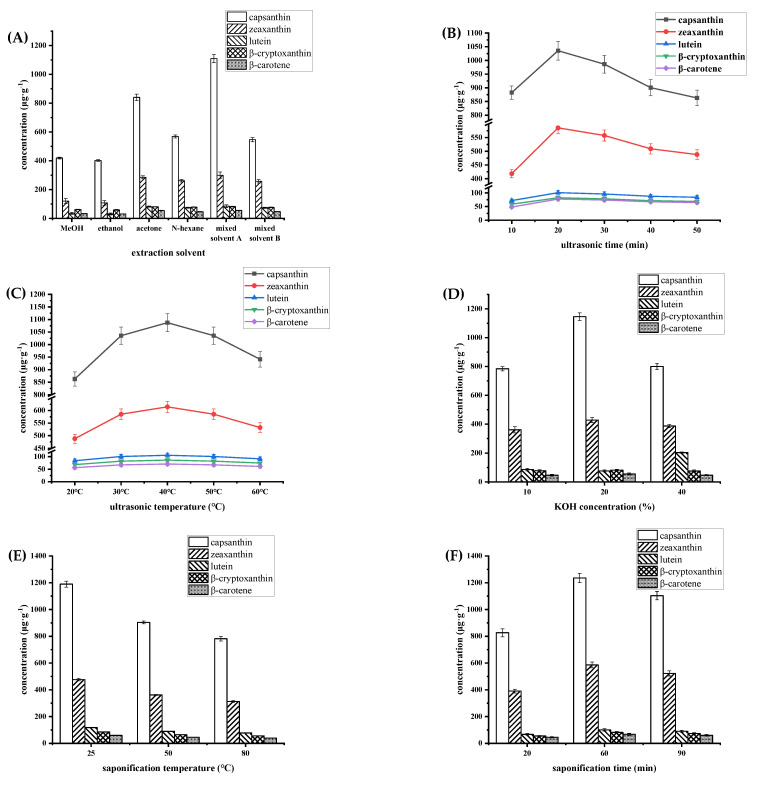
Concentrations of capsanthin, zeaxanthin, lutein, β-cryptoxanthin and β-carotene under different extraction conditions (**A**) extraction solvent, (**B**) ultrasonic time, (**C**) ultrasonic temperature, (**D**) KOH concentration, (**E**) saponification temperature, (**F**) saponification time.

Carotenoid extraction would be affected by improper extraction parameters. As shown in Figure 2, we investigated the optimal extraction methods by single-factor experiments to obtain a higher content of carotenoids. (1) Acetone/anhydrous ether (1:1, *v*/*v*, mixed solvent A) was selected as the extraction solvent because of its higher extraction efficiency, which was consistent with study [29]. In particular, acetone/anhydrous ether (1:1, *v*/*v*, mixed solvent A) provides better extraction of capsanthin, which was most important in red chili peppers. (2) At 20 °C, the extraction effect was at its weakest, likely as a result of the extraction and penetration effect of the extraction solvent being ineffective at such a low temperature [30]. As previously indicated, higher temperatures (>50 °C) could cause the carotenoids to decompose, which is why the content of carotenoids significantly decreased between 40 °C and 60 °C [27]. Additionally, the carotenoid content showed no impact at extraction temperatures of 30 °C, 40 °C and 50 °C, and the content was somewhat better at 40 °C. Finally, 40 °C was decided upon as the extraction temperature. According to the study of Merve Cican [31], β-carotene with higher contents was extracted at 40 °C for 10 to 20 min. (3) Moreover, 20 min was selected as the optimal extraction time, when the carotenoid content was highest and the extraction time was relatively low compared to others [8]. (4) Carotenoids are generally stable in an alkaline environment. Hence, it is advantageous for HPLC analysis to saponify with alkaline solutions following sample extraction to successfully eliminate hydrolyzed carotenoid esters [11]. With an increase in KOH-MeOH concentration, the content of capsanthin and zeaxanthin increased to its highest, at 20% KOH-MeOH, and then decreased; the content of lutein decreased slowly at low alkali concentration and then increased rapidly to be extracted completely when KOH-MeOH was 40%; while the extraction effect of β-cryptoxanthin and β-carotene was not greatly affected by the alkali concentration. Comprehensively, 20% was the optimal KOH-MeOH concentration, where there was the highest content of capsanthin (the main carotenoid). Additionally, in other studies [8,9], capsanthin was extracted from chili peppers using 30% or 40% KOH-MeOH without further optimization, and the targeted peaks of lutein and zeaxanthin were so small that they were challenging to detect [8]. (5) Heating could accelerate the saponification reaction, improve extraction efficiency and reduce carotenoids loss, and 25 °C was selected as the saponification temperature since carotenoids, such as capsanthin and zeaxanthin, were heat-sensitive and decomposed extremely at temperatures of 50 °C to 80 °C. (6) Then, 60 min was determined to be the optimal saponification time when the carotenoid content was at its highest; this was less than that of another study, which used 12 h to soap [8]. As the saponification time increased, the carotenoid content first increased rapidly (20–60 min) and slowly decreased over the course of 60–90 min. This indicates that the saponification is complete at 60 min and the reaction tends to be completed. The degradation of chili pepper carotenoids could be induced by either a higher temperature or a longer heating time.

### 2.3. Methodological Evaluation

Table S2 displays the repeatability test results during four sessions of three groups of mixed standard samples at various concentrations throughout a 14-day period. The RSD% of retention times of capsanthin, zeaxanthin, lutein, β-cryptoxanthin and β-carotene were 0.04~0.22%, 0.05~0.15%, 0.05~0.29%, 0.07~0.17% and 0.03~0.08%, respectively, which were lower than 0.3%. The RSD% of peaks areas of capsanthin, zeaxanthin, lutein, β-cryptoxanthin and β-carotene were 0.16~1.46%, 0.10~0.75%, 0.31~1.39%, 0.30~0.89% and 0.14~1.01%, respectively, which were lower than 1.5%. The results were all lower than those in other studies that determined carotenoids in chili pepper or other vegetables [32,33], which showed that the apparatus and samples were stable among different measurements in a period of time, which was necessary for the validation of the simultaneous HPLC method. 

Each carotenoid was subjected to a linear regression analysis using the standard area value. As shown in Table 1, the *R* coefficients for the calibration curves of the five carotenoids were greater than 0.998, and the concentrations of capsanthin, zeaxanthin, lutein, β-cryptoxanthin, β-carotene standard solutions, measured in the test, all had good linear relationships within the range of 0.1 to 50 mg/L.

The five carotenoids used in the method had LODs and LOQs that varied from 0.020 to 0.063 and 0.067 to 0.209 mg/L, respectively. The LOD_sample_ and LOQ_sample_ of fresh and dried samples of chili peppers and their products are shown in Table 1 for samples weighing 1 g. The LOD_sample_ of fresh chili peppers ranged from 0.497 to 1.565 mg/kg, whereas that of dried, fried, and fermented chili peppers was between 0.500 and 0.875 mg/kg, 0.101 and 0.317 mg/kg, and 0.100 and 0.314 mg/kg, respectively. The LOQ_sample_ ranged from 1.664 to 5.191 mg/kg for fresh chili peppers, 1.675 to 5.225 mg/kg for dried chili peppers, 0.443 to 1.052 mg/kg for fried chili peppers, and 0.334 to 1.042 mg/kg for fermented chili peppers. The LOD and LOQ values were all lower than those in study [34], which proved the superiority of our method with HPLC combined with PDA. For obtaining lower LOD and LOQ, HPLC could be combined with MS, a typical detector with a lower detection limit. For example, the LOD and LOQ of carotenoids in kumquat were 0.07 and 0.22 ppm for β-carotene, 0.1 and 0.33 ppm for β-cryptoxanthin, 0.06 and 0.18 ppm for lutein, 0.08 and 0.3 ppm for zeaxanthin, respectively, obtained by HPLC-DAD-APCI/MS [35].

The five carotenoids were recovered in fresh chili peppers, dried chili peppers, fried chili sauce and fermented chili sauce at rates ranging from 87.80  ±  1.26% to 107.47  ±  3.77%, and more than half of the samples were recovered at 100  ±  5% (Table S3). The samples of dried and fermented processed chili that had fluctuating recoveries may have been affected by the structural composition of the substrate or the influence of food processing technology. The studies of Berhan et al. [33], in determination of lutein, zeaxanthin, α-carotene, β-carotene and β-cryptoxanthin in soybean flour samples by HPLC, achieved sample recoveries from 83.12 to 106.58%, which also indicated that the recoveries in processed samples varied somewhat from 100%.

To further support this new method’s great accuracy, the intraday and interday RSDs, measured by the new method within three days, are shown in Table S3 and ranged from 0.08 to 2.27% and 0.89 to 7.54%, respectively. These values were both lower than 10% [36]. What is more, because of the chili pepper processing method and uneven sampling, the interday RSDs fluctuated slightly significantly more than intraday RSDs, which were also found in other studies [36].

### 2.4. Application of the Method to Fresh Chili Peppers and Chili Pepper Products Analysis

**Figure 3:**
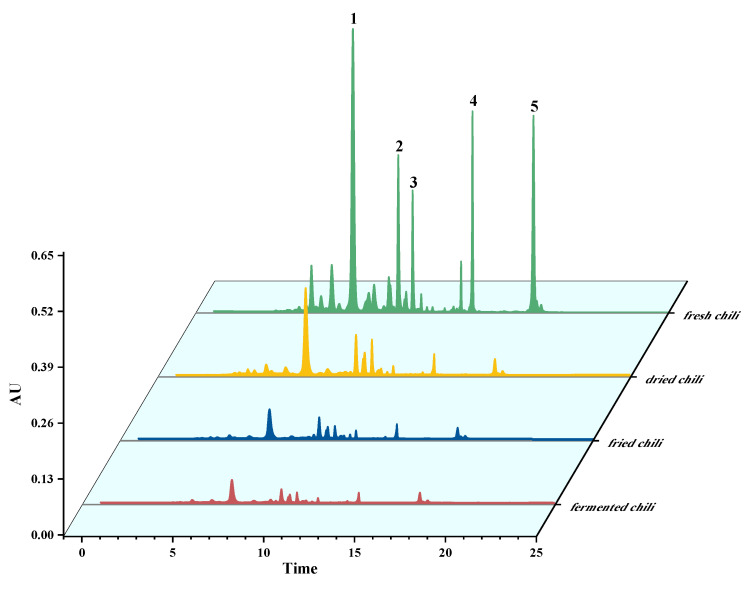
Chromatogram of capsanthin, zeaxanthin, lutein, β-cryptoxanthin and β-carotene.in four kinds of chili peppers and chili pepper products (1: capsanthin, 2: zeaxanthin, 3: lutein, 4: β-cryptoxanthin, 5: β-carotene).

The following four kinds of chili peppers and products were used to validate the improved extraction and HPLC method: fresh chili peppers from Henan, China, dried chili peppers; fried chili sauce; and fermented chili sauce. As shown in Figure 3, the retention times of five carotenoids in samples and standards were identical, and the peaks of each carotenoid were easy to identify in samples thanks to their sharp, symmetrical shapes and high response levels devoid of base substance interference. With strong separation and specificity, the optimized extraction and HPLC method were able to simultaneously determine the capsanthin, zeaxanthin, lutein, β-cryptoxanthin and β-carotene in chili pepper and its products.

**Figure 4:**
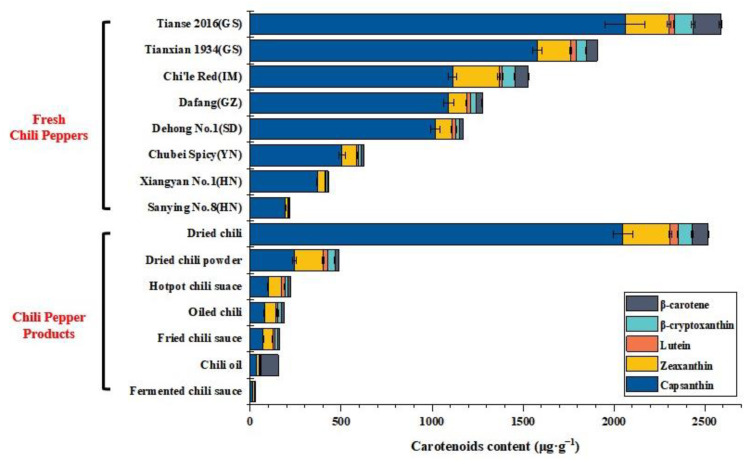
Contents of capsanthin, zeaxanthin, lutein, β-cryptoxanthin and β-carotene in common varieties of fresh chili peppers and chili pepper products (GS, IM, GZ, SD, YN and HN were abbreviations of Gansu, Inner-Monongalia, Guizhou, Shandong, Yunnan and Henan, which were the main provinces of planted chili peppers in China).

Furthermore, the optimized and validated HPLC method was applied for the determination of five carotenoids in nine fresh chili peppers, as shown in Figure 4. Although the contents of the five carotenoids in nine varieties of chili peppers were different, the capsanthin and zeaxanthin accounted for about 70% in red chili peppers, which makes red chili peppers good materials to extract capsanthin. Additionally, because different chili pepper cultivars contain variable contents of carotenoids and capsanthin, they can be used in various processing methods. For example, the capsanthin contents of “Tianse 2016” and “Tianxian 1934” were ranked as the top two; they had capsanthin contents 2.82 and 1.63 times greater than the average capsanthin content in nine samples, respectively, indicating that they are more likely to extract capsanthin. Similarity, the varieties of long and line chili peppers have fewer chili seeds and more chili meat, which was beneficial for extracting more carotenoids without the disturbance of chili seeds. Regarding other varieties of long, line or upright chili peppers with less carotenoids, they can be consumed fresh or made into items such as dried chili peppers and fermented chili sauce. As a result, one of the key criteria for evaluating the effectiveness of chili peppers as extraction materials was their content of carotenoids or capsanthin.

What is more, as shown in Figure 4, the optimized and validated HPLC method was applied for the determination of five carotenoids in seven common chili pepper product samples, including dried chili peppers, dried chili powder, hotpot chili sauce, oiled chili, fried chili sauce, chili oil and fermented chili sauce. The RSDs were 0.81–7.67% (<10%), indicating good accuracy in the results. Additionally, it was discovered that the carotenoid content was dependent on chili pepper varieties and processing methods. Dried chili peppers had more carotenoids because drying was a method that enriched carotenoids by reducing the water in chili peppers. However, dried chili powder’s carotenoid content was lower than that of dried chili peppers because it was processed along with other food such as peppers. Furthermore, oil was used in the processing of oiled chili, fried chili sauce and hotpot chili sauce, which increased the content of carotenoids in those products because carotenoids were easily dissolved in the oil phase [25]. However, the carotenoid content of chili oil, in contrast to oiled chili and other fried chili sauces, was reduced since it was treated with chili peppers and then filtered. As for fermented chili sauce, the long processing time, acid environment and water phase all affected carotenoid stability and dissolution, so the carotenoid content was lowest in fermented chili sauce [25].

## 3. Materials and Methods

### 3.1. Reagents and Materials

Capsanthin (purity 99%) was purchased from Sigma Aldrich (St. Louis, MO, USA). Zeaxanthin, lutein, β-cryptoxanthin and β-carotene (purity 99%) were purchased from Yuanye Bio-Technology (Shanghai, China). HPLC-grade tetrahydrofuran, acetonitrile, methanol (MeOH), acetone, methyl tert-butyl ether (MTBE) and isopropanol were purchased from Thermo Fisher Scientific (Waltham, MA, USA). Analytical-grade N-hexane, MeOH, ethanol, acetone, ethyl acetate, petroleum ether and anhydrous ether were purchased from the Sinopharm Group (Beijing, China). Potassium hydroxide (KOH) was purchased from Lanyi Chemical Company (Beijing, China). Ultrapure water was generated by a Milli-Q integrated water purification system (Millipore, Billerica, MA, USA).

Nine varieties of fresh and red chili peppers, which were selected to be investigated because of the higher and richer carotenoids, were supplied from Comprehensive Experiment Stations of China Agriculture Research System, which were located in Henan, Yunnan, Gansu, Guizhou, Neimen, Shandong (six provinces in China). Dried chili peppers, chili oil, fried chili sauce and fermented chili sauce were all purchased in a market (China).

### 3.2. Apparatus and Software

The weighing experiments were conducted with an analytical scale (BSA 224S-CW, Sartorius, German). Sample pre-treatments were carried out with beater (L18-Y68, Jiuyang Company, Jinan, China), grinder (F203, Krups, Solingen, Germany) and rotary evaporator (Hei-VAP Expert HL/G3, Stinson Technology Company, New York, NY, USA). Sonication treatments were carried out with an ultrasonic bath (PS-40A, Jintan Liangyou Instruments Company, Changzhou, China) of 40 kHz frequency. The centrifuge was Avanti JXN-30 (Beckman Coulter Company, Brea, CA, USA). Wavelength scanning was performed with a UV spectrophotometer (Lambda, PerkinElmer, Woburn, MA, USA). 

HPLC determination was performed with an HPLC system equipped with a quaternary pump, degasser membrane, thermo-stated column compartment, autosampler, a PDA detector and the analytical software, Empower 3.0 (Waters e2695, Milford, MA, USA). The HPLC column was a Spherisorb ODS-2 C18 (250 mm × 4.6 mm, 5 μm) from Agela (Jinan, China). 

### 3.3. Standard Solutions and Samples

Stock solutions of 1 mg/mL of capsanthin, zeaxanthin, lutein, β-cryptoxanthin and β-carotene were prepared by weighing the corresponding mass and subsequently dissolving in anhydrous MeOH to 1 mL. Mixed stock solutions of 100 mg/L were prepared by mixing 100 μL of each standard stock solutions, respectively, and diluting with MeOH. The above solutions were stored below −20 °C and used as soon as possible. Standard solutions were filtered through a 0.22 μm PTFE membrane before the HPLC determination.

One kilogram of fresh chili was washed, diced, and uniformly combined. It was then separated into four equal parts, and two portions (in total around 500 g) were chosen to be mashed into homogeneous paste with a beater; dried chili peppers were ground into a powder (≥40 mesh) with a grinder; chili oil, fried chili sauce and fermented chili sauce were used directly and mixed thoroughly. The above samples were placed in a clean, sealed bag and stored at −20 °C. 

### 3.4. Samples Extraction and Saponification

#### 3.4.1. Optimized Parameters

The standard of the China GB/T 21266-2007 [37], NY/T 1651-2008 [38] and GB 5009.83-2016 [39] methods were used, and modifications were conducted. One gram of each sample (0.2 g chili powder sample) was placed in a centrifuge tube, and then 25 mL acetone and 25 mL anhydrous ether were added and mixed thoroughly. The centrifuge tube was sonicated for 20 min at 40 °C/50 W and centrifuged at 10,000 *g*/4 °C for 5 min to obtain the supernatant. The precipitate was thoroughly mixed with 25 mL of acetone and 25 mL of anhydrous ether before being extracted again. The supernatants were collected together twice.

Then, 50 mL of KOH-MeOH (20%, *w*/*v*) was added to the combined supernatant, shaken well and allowed to rest for 1 h at room temperature (shaken 2~3 times for 30 s each time during resting to ensure sufficient saponification). The saponified solution’s aqueous phase was eliminated, and the organic phase was then rinsed with distilled water until it reached a pH level that was neutral. The saponified solution was then evaporated using a rotary evaporator to dryness at ≤35 °C. Before HPLC analysis, dry material was dissolved with acetone to 5 mL, which was then filtered through a 0.22 μm PTFE membrane into a brown liquid phase vial and stored at −20 °C. Sample solution dilution times were adjusted based on the individual samples so that the sample concentration was located in the middle of a standard curve.

Different extraction parameters were studied, including the solvent selection (MeOH, ethanol, acetone, N-hexane, acetone/anhydrous ether (1:1, *v*/*v*, mixed solvent A), MeOH/ethyl acetate/petroleum ether (1:1:1, *v*/*v*/*v*, mixed solvent B)), extraction temperature (20, 30, 40, 50 and 60 °C), extraction time (10, 20, 30, 40 and 50 min), KOH- MeOH concentration (10%, 20% and 40%), saponification temperature (25, 50 and 80 °C), and saponification time (20, 60 and 90 min).

#### 3.4.2. One Factor Design

Through single-factor experiments, the parameters were optimized by analyzing the separation of peaks, peak areas, response values and the retention times of capsanthin, zeaxanthin, lutein, β-cryptoxanthin and β-carotene in dried chili samples. To rule out the interference of solvent peaks, blank trials were conducted in the interim.

### 3.5. HPLC Conditions

#### 3.5.1. Optimized Parameters

The primary HPLC parameters were as follows: column temperature was 30 °C, detection wavelength was 450 nm, sampling volume was 10 μL, flow rate was 1 mL/min and mobile phases were acetone (solvent A) and water (solvent B), with elution gradients of 0–5 min, 75% A; 5–10 min, 75–95% A; 10–17 min, 95% A; 17–22 min, 95–100% A, 22–27 min, 100–75%A.

The optimization of HPLC parameters was studied, including column temperature (25, 30, 35 °C), flow rate (0.5, 1, 1.5 mL/min), wavelength (400–500 nm), mobile phase A (MeOH, tetrahydrofuran, acetonitrile and acetone), and the elution gradients of mobile phase A (acetone) and mobile phase B (water) (Table S1). 

#### 3.5.2. One Factor Design

The wavelength at which all the five standards (diluted standard stock solution using acetone into 10 mg/L) had greater absorption was selected as the detection wavelength. Other parameters were optimized by analyzing the separation of peaks, peak areas, response values and retention times of capsanthin, zeaxanthin, lutein, β-cryptoxanthin and β-carotene in mixed standard solution (diluted mixed standard stock solution using acetone into 10 mg/L) through single-factor experiments. To rule out the interference of solvent peaks, blank trials were conducted in the interim.

### 3.6. HPLC Determination

The content of each carotenoid (*X_i_*) in the sample was expressed in mg/kg was calculated as following:

Standard curve calibration, by

(1) As=aCs+b

Obtain *a* and b, then

(2) $$C=\frac{\left(A-A0\right)Cs}{aCs+b}$$

The content of each carotenoid *Xi* in the sample was calculated by mass fraction in mg/kg, calculated according to formula as below:

(3) $$X_{i}=\frac{C\times V\times f}{m}\times \frac{1}{1000}$$

Type:

*X_i_*—the content of each carotenoid in the sample, μg/g.
*A*—peak area of each carotenoid in the sample solution.
*A_0_*—peak area of each carotenoid in the sample dilution solution.
*A_s_*—peak area of each carotenoid in the standard solution.
*C_s_*—concentration of each carotenoid in the standard solution, mg/L.
*C*—concentration of each carotenoid in the sample solution, mg/L.
*V*—extract liquid in mL.
*f*—dilution ratio of sample solution.
*m*—mass of sample in g.

Two significant digits were retained for the determination.

### 3.7. Validation Procedures

According to the European Normative, the method was validated by a validation model in accordance with decision 2002/657/EC [40].

For repeatability, mixed standard stock solution was diluted into 1, 5, and 20 mg/L by acetone, which was measured four times over 14 days. Then, the retention times and peak areas were compared among the measurements of four sessions. 

For linearity analysis, mixed standard solutions at concentrations of 0.1, 1, 2, 5, 10, 20, and 50 mg/L were prepared by dilution in acetone. These were injected from a low concentration and followed by analysis and the establishment of standard curves. Linear regression was accomplished by taking the peak area Y as the ordinate and the concentration X as the abscissa. A linear equation was developed by plotting the standard curve of each carotenoid. The standard curves were evaluated based on the linear regression equation and the *R* coefficients for the calibration curves.

For limits of detection and quantification (LODs and LOQs) analysis, mixed standard solutions at concentrations of 0.01, 0.02, 0.05, 0.10, 0.20, 0.50, and 1.00 mg/L were prepared by dilution in acetone. The LODs and LOQs of the method and LOD_sample_ and LOQ_sample_ of the test sample were, respectively, calculated as

LODs = [(*W* × 3) × 100]/[(*S/N*) × 10^−6^] (4)

LOQs = [(*W* × 10) × 100]/[(*S/N*) × 10^−6^] (5)

LOD_sample_ = [(*W* × 3) × *V* × *f* × 100]/[(*S/N*) × *m* × 10^−6^] (6)

LOQ_sample_ = [(*W* × 10) × *V* × *f* × 100]/[(*S/N*) × *m* × 10^−6^] (7)

Type:

*W*—detection of ion concentration, mg/L.
*S/N*—instrument signal to noise ratio.
100—conversion factor.
10^−6^—conversion factor.
*V*—constant volume of sample, mL.
*f*—sample dilution ratio.
*m*—sample weight, g.

For recovery and accuracy analysis, we selected four samples of fresh chili peppers, dried chili peppers, fried chili sauce and fermented chili sauce. The detailed experimental steps were: four sample extracts were diluted with acetone to below the LODs, and then the four dilutions were used to dilute the mixed standard stock solutions to 1, 5 and 20 mg/L. For each level, the same apparatus and operators were used for six repetitions (n = 6) on three consecutive days. Recovery was calculated using the following formula:

(8) $$\mathrm{Recovery}\;\%=\frac{X_{1}-X_{0}}{m}\times 100\%$$

*m*—content of added standard products, mg/L.
*X*_1_—detection of sample extracts added standard products, mg/L.
*X*_0_—detection of sample extracts, mg/L.

### 3.8. Application of the Method to Fresh Chili Peppers and Chili Pepper Products Analysis

The optimized and validated simultaneous HPLC method was applied for the determination of five carotenoids in nine fresh chili peppers and seven common chili pepper products. An in-depth comparison was conducted by analyzing the carotenoid content of samples of fresh chili and chili pepper product samples.

### 3.9. Statistical Analysis

All experiments were conducted in triplicate, and the results were expressed as means ± standard deviation. The results were analyzed using statistical software, and means were accepted as significantly different at a 95% confidence interval (*p* < 0.05). The results were plotted using Origin Pro 2018 software.

## 4. Conclusions

This work reports for the first time the simultaneous determination of five main carotenoids, including capsanthin, zeaxanthin, lutein, β-cryptoxanthin and β-carotene, in chili peppers and their products using a modified HPLC method. The results demonstrated that the HPLC-PDA method, with a broad detection range and low detection limit, which is accurate, stable, reproducible and sensitive, was easy to be applied. With fewer steps and straightforward experimental solvents, it provided an effective and trustworthy method for the qualitative and quantitative analysis of common carotenoids in both fresh chili pepper and its products.

## Figures and Tables

**Table 1:** Validation parameters of the analytical method.

Parameters	Capsanthin	Zeaxanthin	Lutein	β-Cryptoxanthin	β-Carotene
Retention time (min)	7.541	10.187	10.476	14.345	17.873
Standard Curve Equation	Y = 22761X − 24502	Y = 40516X − 2562.6	Y = 124707X − 2148	Y = 74985X − 6336.5	Y = 85211X − 373.54
*R*	0.9985	0.9996	0.9997	0.9999	0.9998
LODs (mg/L)	0.026	0.063	0.020	0.026	0.035
LOQs (mg/L)	0.088	0.209	0.067	0.088	0.118
LOD_sample_ (mg/kg in the fresh chili)	0.646	1.565	0.497	0.646	0.869
LOQ_sample_ (mg/kg in the fresh chili)	2.185	5.191	1.664	2.185	2.931
LOD_sample_ (mg/kg in the dried chili)	0.650	1.575	0.500	0.650	0.875
LOQ_sample_ (mg/kg in the dried chili)	2.200	5.225	1.675	2.200	2.950
LOD_sample_ (mg/kg in the fried chili sauce)	0.131	0.317	0.101	0.131	0.176
LOQ_sample_ (mg/kg in fried chili suace)	0.443	1.052	0.337	0.443	0.594
LOD_sample_ (mg/kg in the fermented chili sauce)	0.130	0.314	0.100	0.130	0.174
LOQ_sample_ (mg/kg in the fermented chili sauce)	0.439	1.042	0.334	0.439	0.588

## Data Availability

The data presented in this study are available in the article and the Supplementary Data.

## Supplementary Materials

The following supporting information can be downloaded at: https://www.mdpi.com/article/10.3390/molecules28052362/s1, Table S1: Five elution gradients of mobile phase A (acetone) and mobile phase B (water); Table S2: Repeatability test results among four sessions of three groups of mixed standard samples of different concentrations in 14 days (n = 6); Table S3: Recovery rate test results of 5 carotenoids in fresh chili peppers, dried chili peppers, fried chili sauce and fermented chili sauce; Figure S1: UV absorption spectra of 5 standards (400~500 nm); Figure S2: Chromatograms of capsanthin, zeaxanthin, lutein, β-cryptoxanthin and β-carotene under different gradient elution (1: capsanthin, 2: zeaxanthin, 3: lutein, 4: β-cryptoxanthin, 5: β-carotene; a: method 1, b: method 2, c: method 3, d: method 4, e: method 5, f: method 6); Figure S3: Chromatograms of capsanthin, zeaxanthin, lutein, β-cryptoxanthin and β-carotene eluted by different mobile phases (a: acetone–water, b: MeOH–water, c: b: tetrahydrofuran–water, d: acetonitrile–water); Figure S4: Chromatograms of capsanthin, zeaxanthin, lutein, β-cryptoxanthin and β-carotene eluted at different flow rates (1: capsanthin, 2: zeaxanthin, 3: lutein, 4: β-cryptoxanthin, 5: β-carotene; a: 0.5 mL/min, b: 1.0 mL/min, c: 1.5 mL/min); Figure S5: Chromatograms of capsanthin, zeaxanthin, lutein, β-cryptoxanthin and β-carotene eluted at different column temperatures (1: capsanthin, 2: zeaxanthin, 3: lutein, 4: β-cryptoxanthin, 5: β-carotene; a: 25 °C, b: 30 °C, c: 35 °C)

## Sample Availability

Samples of the compounds are not available from the authors.
