# Supplementary material for: Development of an HPLC-PDA Method for the Determination of Capsanthin, Zeaxanthin, Lutein, β-Cryptoxanthin and β-Carotene Simultaneously in Chili Peppers and Products
Source: Molecules. 2023 Mar 3;28(5):2362. doi: 10.3390/molecules28052362 (PMC10005789; doi:10.3390/molecules28052362)
Supplement: Supplementary file 1 [file molecules-28-02362-s001.zip › molecules-2233785-supplementary.pdf]

Table S1 Five elution gradients of mobile phase A (acetone) and mobile phase B (water)

Method 1

| Time / min | A / % | B / % |
|---|---|---|
| 0 | 30 | 70 |
| 5 | 30 | 70 |
| 10 | 85 | 15 |
| 17 | 85 | 15 |
| 22 | 100 | 0 |
| 27 | 30 | 70 |

Method 2

| Time / min | A / % | B / % |
|---|---|---|
| 0 | 55 | 45 |
| 5 | 65 | 35 |
| 7 | 75 | 25 |
| 10 | 85 | 15 |
| 16 | 55 | 45 |

Method 3

| Time / min | A / % | B / % |
|---|---|---|
| 0 | 55 | 45 |
| 5 | 55 | 35 |
| 15 | 75 | 25 |
| 22 | 85 | 15 |
| 27 | 55 | 45 |

Method 4

| Time / min | A / % | B / % |
|---|---|---|
| 0 | 90 | 10 |
| 2 | 90 | 10 |
| 10 | 80 | 20 |
| 20 | 30 | 70 |
| 22 | 0 | 100 |
| 27 | 90 | 10 |

Method 5

Isocratic elution, 90% A.

Method 6

| Time / min | A / % | B / % |
|---|---|---|
| 0 | 75 | 25 |
| 5 | 75 | 25 |
| 10 | 95 | 5 |
| 17 | 95 | 5 |
| 22 | 100 | 0 |
| 27 | 75 | 25 |

Table S2 Repeatability test results among four sessions of three groups of mixed standard samples of different concentrations in 14 days (n=6)

| | Storage time / d | Capsanthin | | Zeaxanthin | | Lutein | | β-cryptoxanthin | | β-carotene | |
|---|---|---|---|---|---|---|---|---|---|---|---|
| | | RSD% of retention time | RSD% of peak area | RSD% of retention time | RSD% of peak area | RSD% of retention time | RSD% of peak area | RSD% of retention time | RSD% of peak area | RSD% of retention time | RSD% of peak area |
| 1 μg/mL | 0 | 0.04 | 1.10 | 0.15 | 0.48 | 0.29 | 1.13 | 0.11 | 0.37 | 0.07 | 0.33 |
| | 3 | 0.22 | 0.93 | 0.10 | 0.67 | 0.08 | 1.17 | 0.17 | 0.37 | 0.08 | 0.14 |
| | 7 | 0.17 | 0.39 | 0.14 | 0.75 | 0.15 | 1.23 | 0.08 | 0.40 | 0.08 | 0.37 |
| | 14 | 0.07 | 0.33 | 0.14 | 0.28 | 0.08 | 1.39 | 0.11 | 0.45 | 0.07 | 0.39 |
| 2 μg/mL | 0 | 0.12 | 0.26 | 0.05 | 0.35 | 0.05 | 0.94 | 0.10 | 0.30 | 0.05 | 0.46 |
| | 3 | 0.19 | 0.19 | 0.12 | 0.34 | 0.15 | 0.47 | 0.09 | 0.36 | 0.05 | 0.46 |
| | 7 | 0.22 | 0.23 | 0.06 | 0.32 | 0.08 | 0.97 | 0.08 | 0.39 | 0.08 | 0.61 |
| | 14 | 0.16 | 0.16 | 0.11 | 0.20 | 0.16 | 0.97 | 0.09 | 0.36 | 0.08 | 0.64 |
| 5 μg/mL | 0 | 0.07 | 0.47 | 0.10 | 0.15 | 0.08 | 0.54 | 0.12 | 0.63 | 0.06 | 0.55 |
| | 3 | 0.08 | 1.46 | 0.06 | 0.12 | 0.13 | 0.43 | 0.09 | 0.50 | 0.05 | 0.65 |
| | 7 | 0.15 | 1.43 | 0.09 | 0.13 | 0.07 | 0.59 | 0.07 | 0.89 | 0.03 | 0.75 |
| | 14 | 0.10 | 1.09 | 0.13 | 0.10 | 0.12 | 0.31 | 0.10 | 0.51 | 0.06 | 1.01 |

Table S3 Recovery rate test results of 5 carotenoids in fresh chili peppers, dried chili peppers, fried chili sauce and fermented chili sauce

| Sample | Compound name | 1 mg/kg | | | 5 mg/kg | | | 10 mg/kg | | |
|---|---|---|---|---|---|---|---|---|---|---|
| | | Average recovery rate% | Intraday RSD% | Interday RSD% | Average recovery rate% | Intraday RSD% | Interday RSD% | Average recovery rate% | Intraday RSD% | Interday RSD% |
| Fresh chili peppers | Capsanthin | 99.28±3.70 | 1.01 | 3.72 | 100.96±6.39 | 0.88 | 6.33 | 99.60±6.41 | 0.78 | 6.44 |
| | Zeaxanthin | 99.60±6.41 | 0.98 | 1.25 | 100.73±1.25 | 0.45 | 2.08 | 102.28±2.12 | 0.60 | 3.50 |
| | Lutein | 91.51±1.22 | 0.48 | 1.33 | 94.05±1.95 | 1.07 | 2.07 | 95.90±4.29 | 0.77 | 4.47 |
| | β-cryptoxanthin | 95.51±3.30 | 0.75 | 3.46 | 96.98±4.25 | 1.10 | 4.38 | 98.48±3.02 | 2.27 | 3.06 |
| | β-carotene | 98.35±4.78 | 0.57 | 4.86 | 97.80±4.02 | 0.62 | 4.11 | 99.00±2.16 | 1.13 | 2.18 |
| Dried chili peppers | Capsanthin | 90.35±2.25 | 0.93 | 2.48 | 87.80±1.26 | 0.24 | 1.44 | 101.35±4.34 | 0.63 | 4.29 |
| | Zeaxanthin | 102.07±3.17 | 0.88 | 3.11 | 97.07±1.33 | 1.01 | 1.37 | 98.10±3.26 | 0.65 | 3.32 |
| | Lutein | 99.77±2.28 | 0.64 | 2.28 | 100.14±2.29 | 0.21 | 2.29 | 97.32±2.98 | 0.87 | 3.07 |
| | β-cryptoxanthin | 96.76±4.86 | 0.45 | 5.02 | 95.24±2.88 | 0.09 | 3.02 | 98.38±2.49 | 0.89 | 2.52 |
| | β-carotene | 97.22±3.39 | 0.98 | 3.49 | 95.00±1.57 | 0.49 | 1.65 | 99.03±2.01 | 0.97 | 2.04 |
| Fried chili sauce | Capsanthin | 92.47±2.52 | 1.47 | 2.72 | 98.69±5.74 | 0.43 | 5.81 | 107.47±3.77 | 0.64 | 3.51 |
| | Zeaxanthin | 97.44±3.35 | 0.89 | 3.44 | 100.56±1.80 | 0.20 | 1.79 | 99.14±4.10 | 0.38 | 4.13 |
| | Lutein | 96.91±2.67 | 0.38 | 2.75 | 99.56±2.38 | 1.26 | 2.39 | 99.32±6.62 | 0.08 | 6.66 |
| | β-cryptoxanthin | 98.13±6.63 | 0.86 | 6.76 | 98.80±0.88 | 1.18 | 0.89 | 97.61±4.34 | 0.12 | 4.44 |
| | β-carotene | 96.61±5.16 | 1.14 | 5.34 | 97.60±3.72 | 1.25 | 3.82 | 97.56±3.32 | 1.08 | 3.40 |
| Fermented chili sauce | Capsanthin | 103.31±1.99 | 1.86 | 1.93 | 99.08±7.47 | 0.84 | 7.54 | 102.67±5.59 | 0.27 | 5.45 |
| | Zeaxanthin | 103.19±5.43 | 0.99 | 5.26 | 97.20±3.63 | 0.99 | 3.74 | 97.14±2.25 | 0.29 | 2.31 |
| | Lutein | 98.38±3.35 | 0.38 | 3.40 | 99.53±4.64 | 1.19 | 4.66 | 98.90±3.27 | 1.52 | 3.30 |
| | β-cryptoxanthin | 98.93±2.48 | 0.86 | 2.50 | 101.40±5.26 | 0.52 | 5.19 | 99.02±5.18 | 0.53 | 5.23 |
| | β-carotene | 97.97±2.86 | 1.09 | 2.92 | 97.61±2.37 | 1.04 | 2.42 | 95.43±1.66 | 2.18 | 1.74 |

(Note: Average recovery rate% was the average of 18 samples (n=6 / day) in three days which was expressed as mean ± standard deviation.)

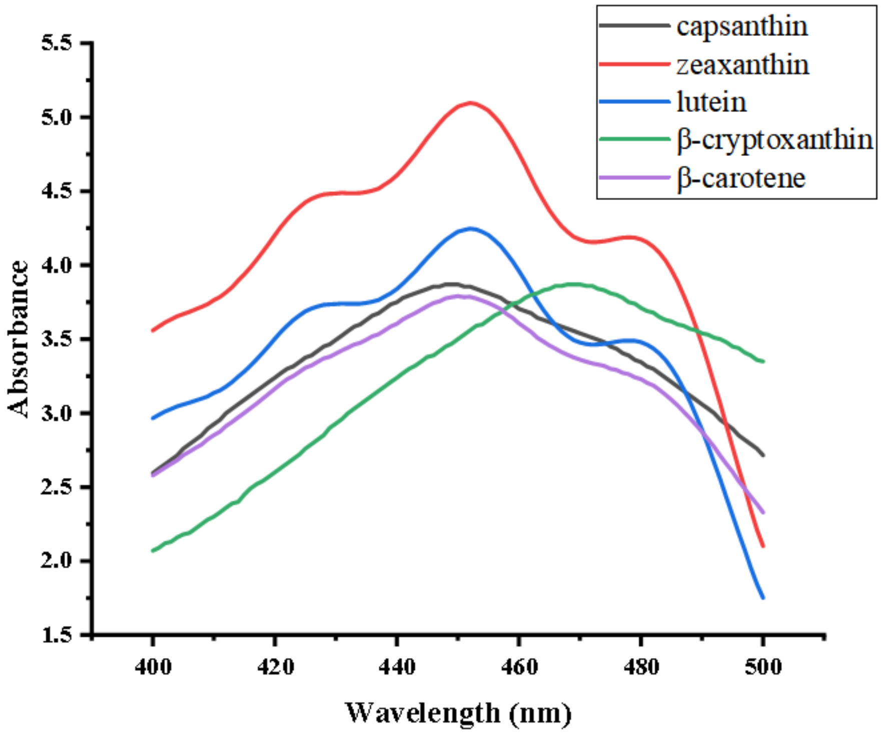


Figure. S1. UV absorption spectra of 5 standards in acetone, obtained by the DAD detector (400~500 nm).

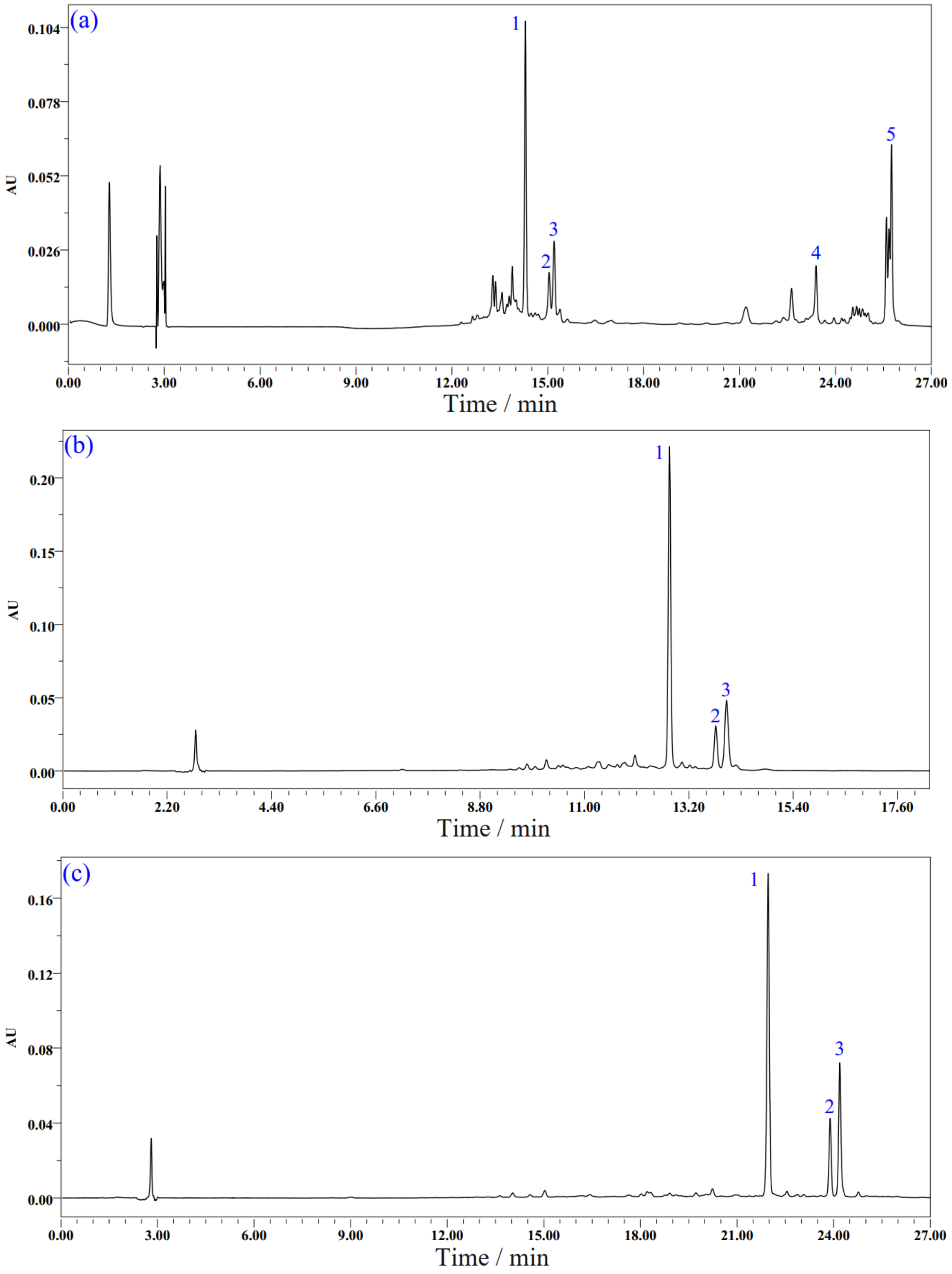
(a)
1
2
3
4
5
0.104
0.078
0.052
0.026
0.000
AU
0.00
3.00
6.00
9.00
12.00
15.00
18.00
21.00
24.00
27.00
Time / min
(b)
1
2
3
0.20
0.15
0.10
0.05
0.00
AU
0.00
2.20
4.40
6.60
8.80
11.00
13.20
15.40
17.60
Time / min
(c)
1
2
3
0.16
0.12
0.08
0.04
0.00
AU
0.00
3.00
6.00
9.00
12.00
15.00
18.00
21.00
24.00
27.00
Time / min

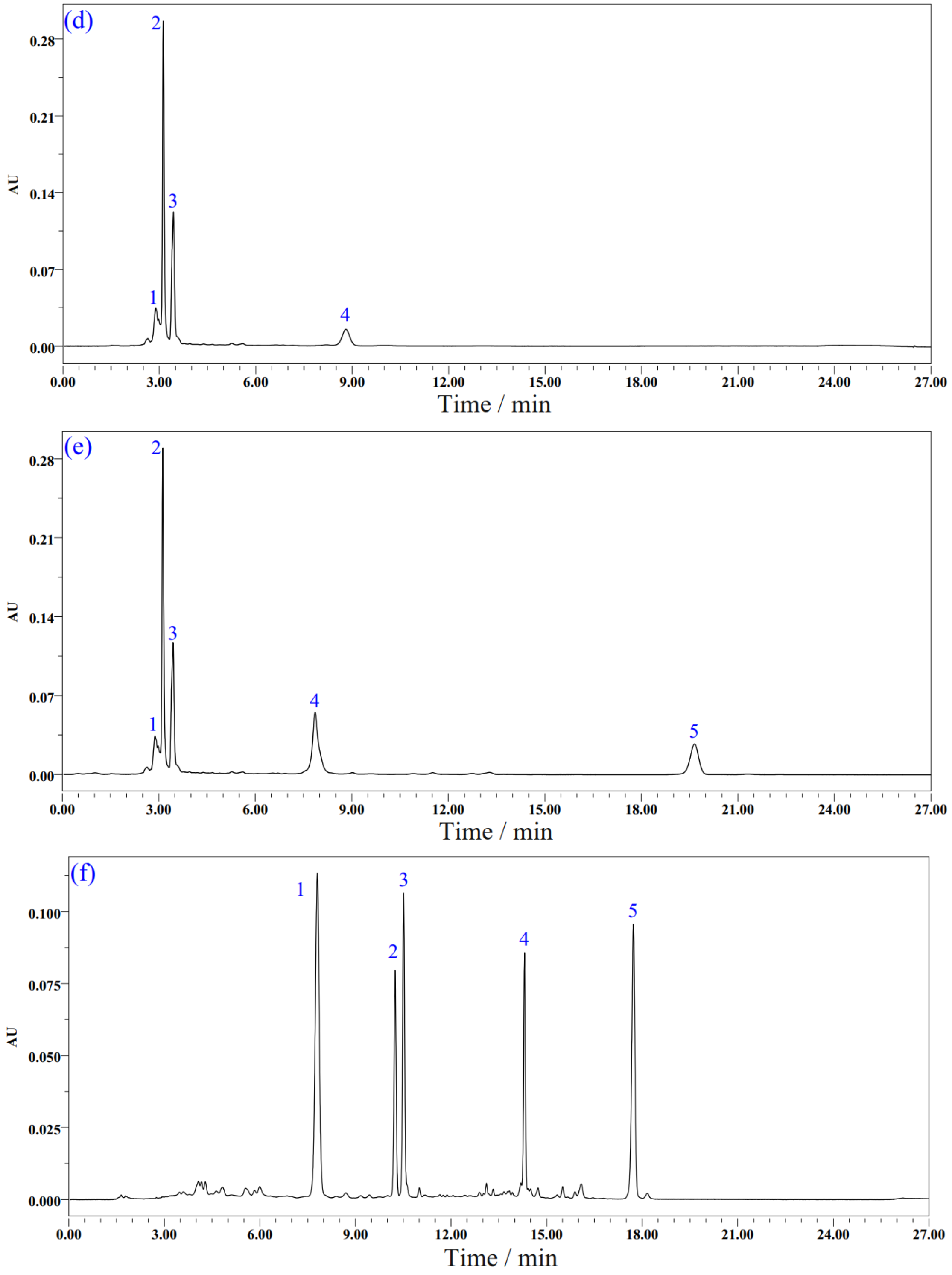


Figure. S2. Chromatograms of capsanthin, zeaxanthin, lutein, β-cryptoxanthin and β-carotene under different gradient elution

(1: capsanthin, 2: zeaxanthin, 3: lutein, 4: β-cryptoxanthin, 5: β-carotene;

a: method 1, b: method 2, c: method 3, d: method 4, e: method 5, f: method 6)

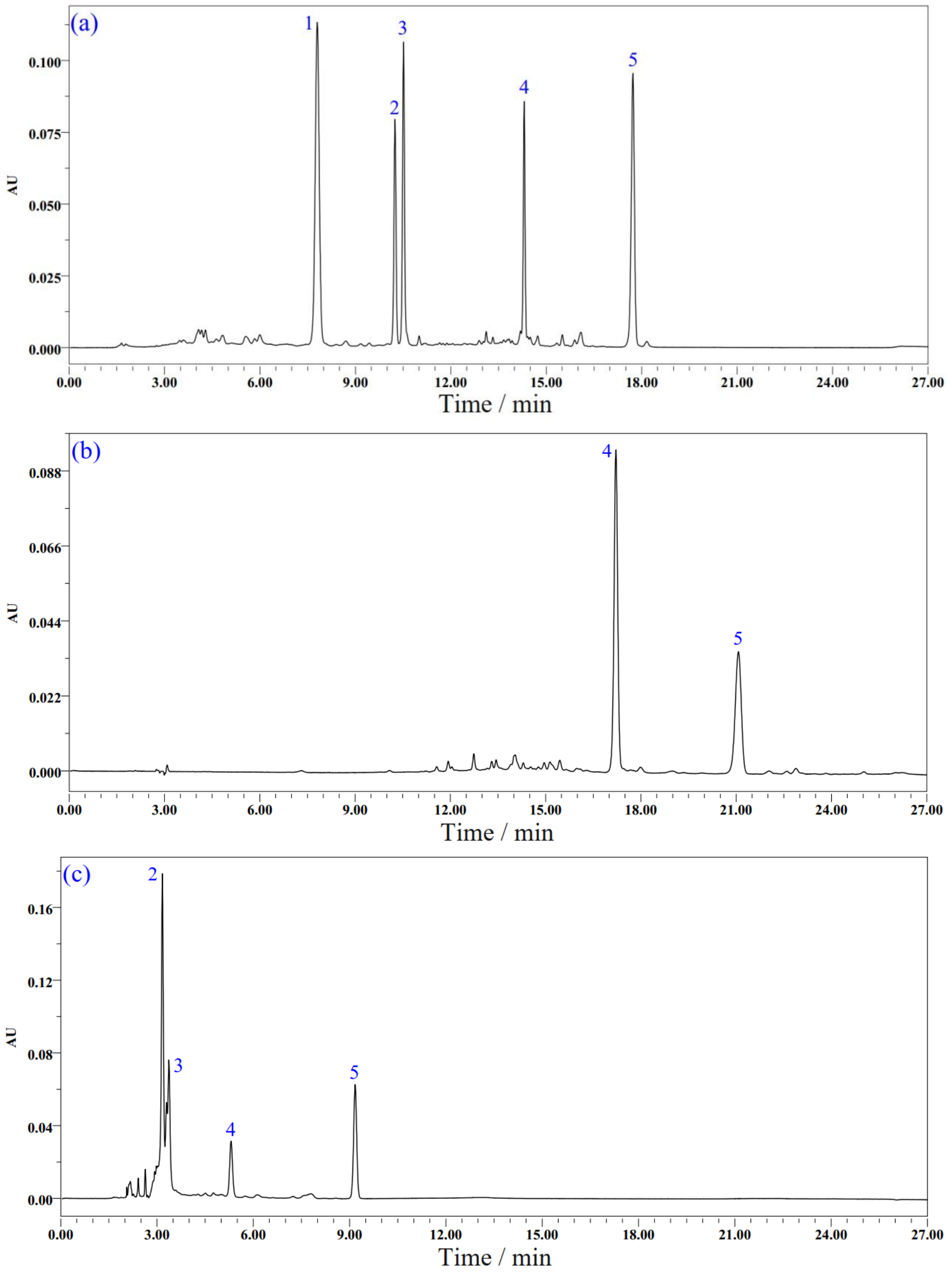
(a)
AU
0.100
0.075
0.050
0.025
0.000
1
2
3
4
5
0.00
3.00
6.00
9.00
12.00
15.00
18.00
21.00
24.00
27.00
Time / min
(b)
AU
0.088
0.066
0.044
0.022
0.000
4
5
Time / min
(c)
AU
0.16
0.12
0.08
0.04
0.00
2
3
4
5
Time / min

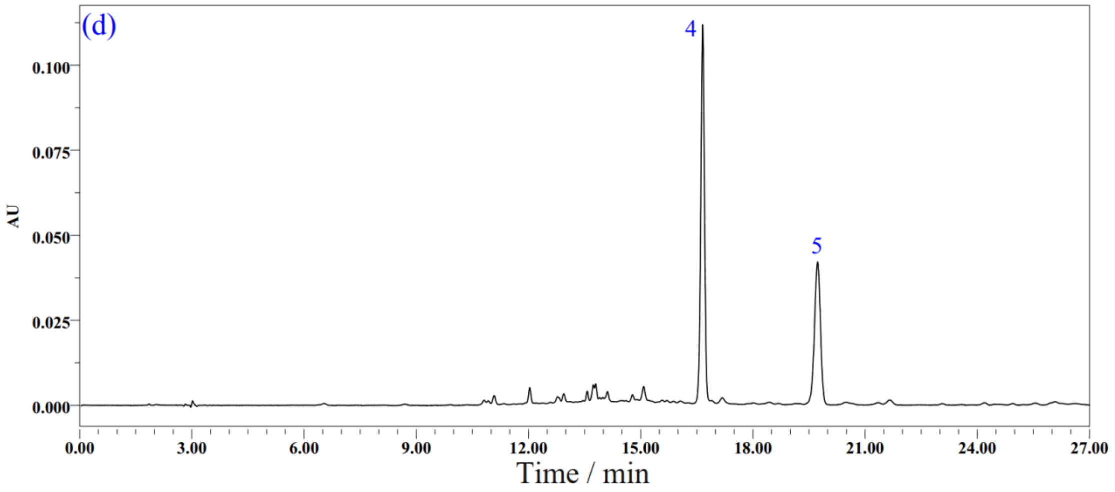


Figure. S3. Chromatograms of capsanthin, zeaxanthin, lutein, β-cryptoxanthin and β-carotene eluted by different mobile phases(a: acetone-water, b: MeOH-water, c: b: tetrahydrofuran-water, d: acetonitrile-water)

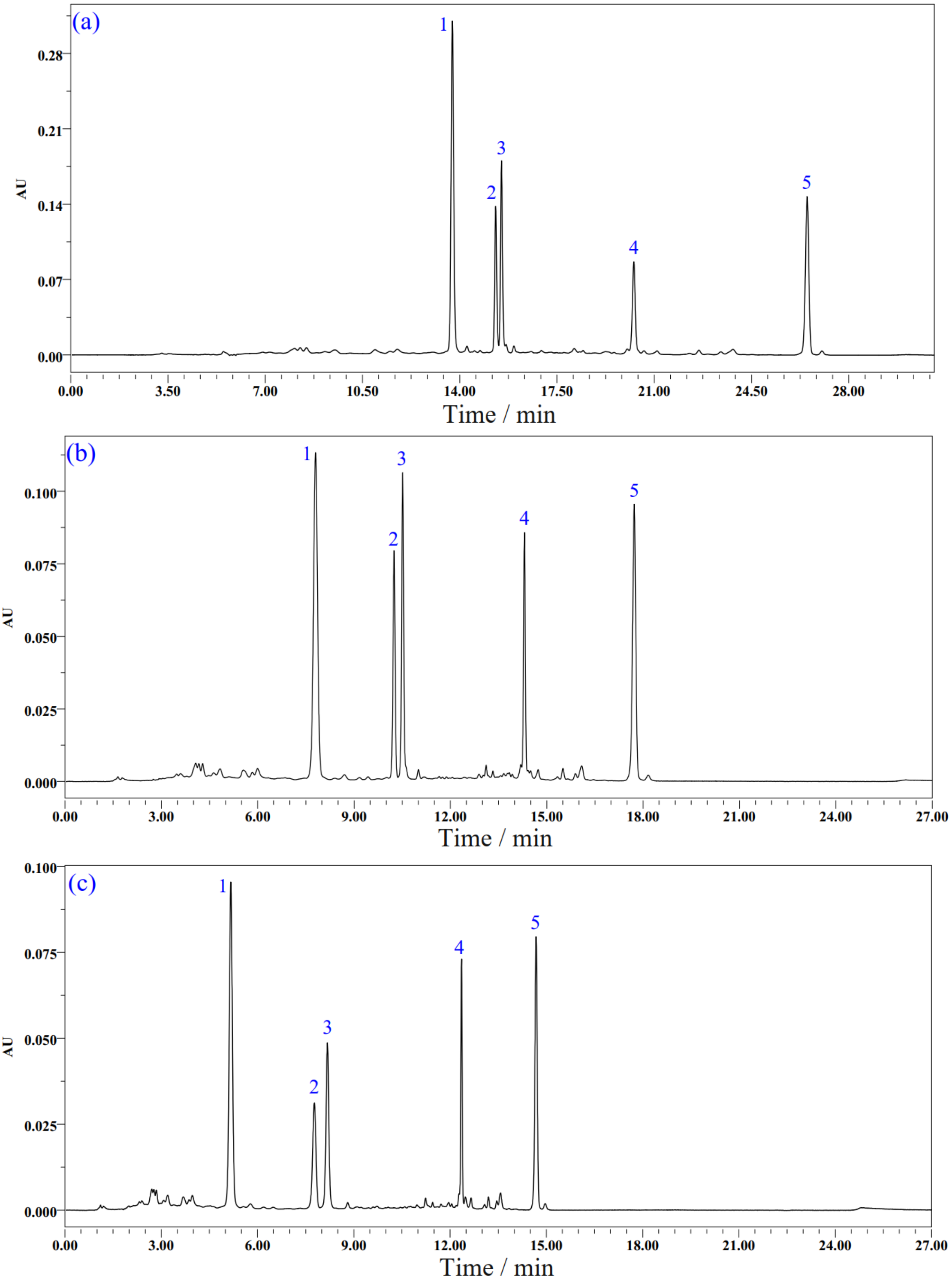


Figure. S4. Chromatograms of capsanthin, zeaxanthin, lutein, β-cryptoxanthin and β-carotene eluted at different flow rates(1: capsanthin, 2: zeaxanthin, 3: lutein, 4: β-cryptoxanthin, 5: β-carotene; a: 0.5 mL/min, b: 1.0 mL/min, c: 1.5 mL/min)

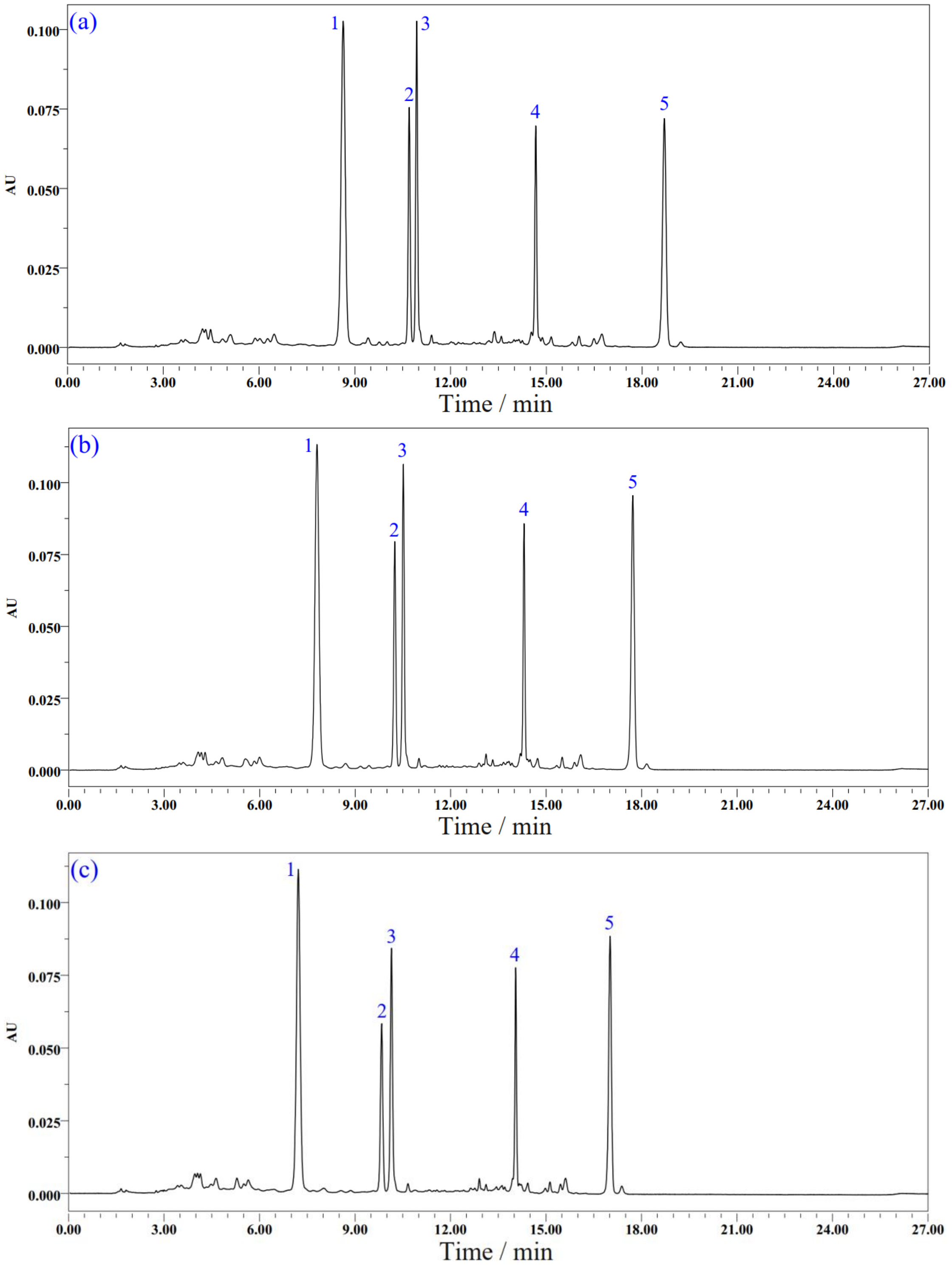


Figure.S5. Chromatograms of capsanthin, zeaxanthin, lutein, β-cryptoxanthin and β-carotene eluted at different column temperatures(1: capsanthin, 2: zeaxanthin, 3: lutein, 4: β-cryptoxanthin, 5: β-carotene; a: 25 °C, b: 30 °C, c: 35 °C)
